# Trans-Allelic Model for Prediction of Peptide:MHC-II Interactions

**DOI:** 10.3389/fimmu.2018.01410

**Published:** 2018-06-20

**Authors:** Abdoelnaser M. Degoot, Faraimunashe Chirove, Wilfred Ndifon

**Affiliations:** ^1^African Institute of Mathematical Sciences (AIMS), Muizenberg, South Africa; ^2^School of Mathematics, Statistics and Computer Science, University of KwaZulu-Natal, Pietermaritzburg, South Africa; ^3^DST-NRF Centre of Excellence in Mathematical and Statistical Sciences (CoE-MaSS), Gauteng, South Africa

**Keywords:** major histocompatibility complex (MHC), modeling peptide–MHC-II interactions, antigen presentation, machine learning, inverse statistical mechanics

## Abstract

Major histocompatibility complex class two (MHC-II) molecules are trans-membrane proteins and key components of the cellular immune system. Upon recognition of foreign peptides expressed on the MHC-II binding groove, CD4^+^ T cells mount an immune response against invading pathogens. Therefore, mechanistic identification and knowledge of physicochemical features that govern interactions between peptides and MHC-II molecules is useful for the design of effective epitope-based vaccines, as well as for understanding of immune responses. In this article, we present a comprehensive trans-allelic prediction model, a generalized version of our previous biophysical model, that can predict peptide interactions for all three human MHC-II loci (HLA-DR, HLA-DP, and HLA-DQ), using both peptide sequence data and structural information of MHC-II molecules. The advantage of this approach over other machine learning models is that it offers a simple and plausible physical explanation for peptide–MHC-II interactions. We train the model using a benchmark experimental dataset and measure its predictive performance using novel data. Despite its relative simplicity, we find that the model has comparable performance to the state-of-the-art method, the NetMHCIIpan method. Focusing on the physical basis of peptide–MHC binding, we find support for previous theoretical predictions about the contributions of certain binding pockets to the binding energy. In addition, we find that binding pocket *P*5 of HLA-DP, which was not previously considered as a primary anchor, does make strong contribution to the binding energy. Together, the results indicate that our model can serve as a useful complement to alternative approaches to predicting peptide–MHC interactions.

## 1 Introduction

Major histocompatibility complex class two (MHC-II) molecules are surface proteins that exist on the membrane of antigen presenting cells (APCs) such as macrophages, dendritic cells, and B cells. They bind short peptide fragments derived from exogenous proteins and present them to *CD*4^+^ helper-T cells. Upon the recognition of foreign peptides presented by MHC-II molecules, the helper-T cells (precisely speaking, *CD*4^+^ effector T cells) will initiate proper adaptive immune responses, including enabling sufficient maturation of B cells and cytotoxic *CD*8^+^ T cells (1). Therefore, the binding of peptide to MHC-II molecules is considered to be a fundamental and pre-requisite step in the initiation of adaptive immunity (2, 3). As such, mechanistic identification of the basic determinants of peptide–MHC-II interactions presents potential for understanding the immune system’s mechanisms and improving the process of designing peptide- and protein-based vaccines.

MHC genes for humans, referred to as human leukocyte antigen (HLA), are among the most polymorphic genetic elements found within a long continuous stretch of DNA on chromosome 6 (4). Such high polymorphism reflects the immense contribution of MHC molecules to the adaptive immune system and underpins their capacity to recognize a wide range of pathogens. Nonetheless, some viruses, such as hepatitis C, avian/swine influenza, and human immunodeficiency virus (HIV), undergo extensive mutations that allow them to partially escape recognition by the MHC molecules (5). MHC genes can be divided into HLA class I, II, and III. Loci corresponding to HLA class I are A, B, and C; HLA class II loci are DP, DQ, and DR; HLA class III genes encode for several other immune-related proteins and provide support for the former two classes (1, 4).

MHC-II molecules account for the likelihood of success of organ transplantation, and there are well-established associations between many disorders and particular classes of MHC-II molecules. These include the contribution of HLA-DQ genes to insulin-dependent diabetes (6); HLA-DR genes to multiple sclerosis; and narcolepsy (7) along with other autoimmune diseases resulting from degeneracy and misregulation in the process of peptide presentation (8). Moreover, genetic and epidemiological data have implicated MHC-II molecules in susceptibility to many infectious diseases such as HIV/AIDS, malaria (9), and cancer (10).

Experimental assays for prediction of peptide–MHC-II interactions are often faced with important obstacles, including substantial resources needed for laboratory work, high time, and labor demands. This is the case in particular, for experimental work aimed at finding out which promiscuous epitopes bind to specific MHC molecules, a necessary step in the design of peptide-based vaccines which protect against a broad range of pathogen variants. Computational methods, which are more efficient and less costly than biological assays, have been employed to complement these assays. Due to advances in sequencing technologies, immunological data have grown at an unprecedented pace and continue to accrue. This has been exploited in systematic computational analyses of genomes of multiple pathogens to determine which subunits might induce a potent immune response. The results have been the design and development of new vaccine candidates against HIV, influenza, and other hyper-variable viruses (11). Use of computational methods has significantly reduced experimental effort and costs by up to 85% (12).

Many immunoinformatics methods for prediction of peptide–MHC interactions, for both class I and II, have been developed based on machine learning approaches such as simple pattern motif (13), support vector machine (SVM) (14), hidden Markov model (HMM) (15), neural network (NN) models (16–18), quantitative structure–activity relationship (QSAR) analysis (19), structure-based methods, and biophysical methods (2, 20, 21; Degoot et al., unpublished). These methods can be divided into two categories, namely, intra-allele (allele-specific) and trans-allele (pan-specific) methods. Intra-allelic methods are trained for a specific MHC molecule on a limited set of experimental peptide-binding data and applied for prediction of peptides binding to that molecule. Because of the extreme polymorphism of MHC molecules, the existence of thousands of allele variants, combined with the lack of sufficient experimental binding data, it is impossible to build a prediction model for each allele. Thus, trans-allele and general purpose (22) methods such as *MULTIRTA* (2), *NetMHCIIpan* (18), and *TEPITOPEpan* (23) have been developed using richer peptide-binding data expanding over many alleles or across species (18). Similar methods for MHC-I are also available such as NetMHCpan (24, 25) and KISS (26).

The trans-allelic models are often designed to extrapolate either structural similarities or shared physicochemical binding determinants among HLA genes, to predict affinities for alleles that are not part of the training dataset. These models generally have better predictive performance for new alleles and a wide range of potential applications compared with the intra-allelic models.

Most of the existing trans-allelic models for MHC-II are extended versions of their earlier intra-allelic counterparts: TEPITOPEpan (23) was extended from *TEPITOPE* (27); *MULTIRTA* (2) evolved from *RTA* (20); and the series of *NetMHCIIpans* (1.0, 2.0, 3.0, and 3.1) (17, 18, 28, 29) were generalized from the NN align (30) method. In the same vein, in this article, we present a trans-allele method, an extension of our previous method (Degoot et al., unpublished), for prediction of peptide-HLA class II interactions based on biophysical ideas.

The remarkable strength of the method presented here over other existing advanced data-driven approaches is its physical basis. We formulate the process of binding affinity between peptide and MHC-II molecule as an inverse problem of statistical physics. From the observable macroscopic (bound and unbound) states of experimental data, we compute the microscopic parameters (Hamiltonians for amino acid residues involved in the interaction) that govern the system. In fact, many problems in computational biology can be solved in such a way (31, 32), taking advantage of the availability of vast amount of genomic data and high resolution structural information. Solutions obtained using this approach are more plausible and physically interpretable than those obtained using mere sequence-based methods (2; Degoot et al., unpublished). In addition, because sparsity is a hallmark feature of biological processes, we adjust the model’s parameters via incorporating an 𝖫_𝟣_ regularization term into the model. The 𝖫_𝟣_ constraint, commonly named *Lasso*, encourages sparsity and improves the predictive performance of the model on novel data.

The rest of this article is organized as follows: in Section 2.1, we describe the idea of MHC-II polymorphic residue groups, which is employed to capture structure similarity among MHC-II alleles. In Section 2.2, we define our methodology and formulate the learning function. After that we briefly describe the benchmark dataset used to test the predictive performance of the model in Section 2.3 and present the results in Section 3. Finally, in Section 3.3, we summarize and discuss our results and compare our method with the state-of-the art method.

## 2 Materials and Methods

### 2.1 MHC-II Polymorphic Residue Groups

Crystal structures revealed that an MHC molecule is a combination of two domains, an *α* helix and a *β* sheet, linked together to form a Y-shaped groove which is used to locate peptides, and both domains equally contribute to the binding affinity. For HLA-I molecules, the *β* domain is largely conserved, and variation occurs mostly in the *α* domain. On the other hand, polymorphism occurs in both domains of HLA-II molecules; except for HLA-DR alleles, where the variation takes place in the *β* domain. In addition, the peptide-binding groove of the HLA-II is open at both ends, which allows binding peptides of variable lengths (33), ranging from 9 to 30 amino acid residues, or even an entire protein (29, 34). This is in contrast to the peptide-binding groove of the HLA-I alleles, which accommodate only short peptides of lengths ranging from 8 to 11 amino acids. This flexible constraint on peptide lengths together with its immense polymorphism, contribute to a lower predictive performance of computational methods for peptide–MHC-II interactions compared with MHC-I methods (2, 22).

The notion of MHC polymorphic residue groups, introduced by Bordner and Mittelmann (2), is based on a simple observation of an intrinsic (independent of peptide) feature of the MHC-II binding groove. Although a peptide could bind to an MHC-II molecule in various registers, due to the open-ended nature of the MHC-II binding groove, the strength of the binding affinity is primarily determined by 9 residues occupying the binding groove pockets. Interestingly, most of polymorphism in MHC-II genes occurs at these binding pockets (see the discussion in Section 3.3).

From the limited available crystallographic structural data of peptide–MHC-II complexes for a few MHC-II molecules from the Protein Data Bank (PDB) (35) (summarized in Table S1 in Supplementary Material), sets of important positions for the polymorphic residues in the binding groove that contact one or more peptide-binding cores and are within a distance of not more than 4 Å (2, 18, 36) in one or more of the MHC-II complex structures can be extracted. Then, by extrapolating the similarities among MHC molecules, their corresponding residues in different genes are determined using multiple sequence analysis (MSA) (37). Exploiting the fact that HLA-DR alleles are polymorphic only in the *β* domain and have the same *α* domain, the polymorphic residue groups for HLA-DR are extracted from its *β* domain sequences. Similarly, assuming sufficiency of the *β* domains for predicting MHC–peptide binding preferences (2) and for the sake of simplicity of the model, residue groups for HLA-DP and HLA-DQ were also extracted from the *β* domain.

Next, the set of polymorphic residues that always co-occur at the specified positions are clustered into the same group. The rationale of clustering polymorphic residue groups, rather than individual residues, is to avoid over-parametrization of the model. Table S2 in Supplementary Material shows such polymorphic residue groups for HLA-DRB, HLA-DP, and HLA-DQ alleles, assembled by the procedures described earlier.

### 2.2 Trans-Allele Model

In our previous intra-allele model (Degoot et al., unpublished) the probability of peptide 𝖯^(𝗄)^ to bind an MHC molecule 𝖬^(𝖳(𝗄))^ was computed as follows:

(1) $$\pi \left({\text{P}}^{\left(\text{k}\right)},{\text{M}}^{\left(\text{T}\left(\text{k}\right)\right)}\right)=\frac{1}{1+e^{\delta {\text{E}}^{\left(\text{k}\right)}}},$$

where *δ*𝖤^(𝗄)^ is the change in binding energy in terms of the sum of the differences of first- and second-order Hamiltonians between the bound and unbound states. Specifically, *δ*𝖤^(𝗄)^ is given by the following equation:

(2) $$\delta {\text{E}}^{\left(\text{k}\right)}=\underset{\text{first-order Hamiltonians}}{\underset{︸}{\underset{i=1}{\overset{\left|{\text{P}}^{\left(\text{k}\right)}\right|}{\sum }}\;\delta H^{\left(1\right)}\left({\text{a}}_{i}\right)+\underset{i=1}{\overset{9}{\sum }}\;\delta H^{\left(1\right)}\left({\text{b}}_{i}\right)+\delta S}}+\overset{\text{per residue-residue interactions}}{\overset{︷}{\underset{\text{second-order Hamiltonians}}{\underset{︸}{\underset{i=1}{\overset{\left|{\text{P}}^{\left(\text{k}\right)}\right|}{\sum }}\;\underset{j=1}{\overset{9}{\sum }}\;\underset{r=1}{\overset{\text{R}}{\sum }}\;\delta H^{\left(2\right)}\left({\text{a}}_{\mathrm{ir}}^{\left(k\right)},{\text{b}}_{j}\right)}}}},$$

in which |𝖯^(𝗄)^| is the length of peptide 𝗄, 𝖱 is the number of all possible configurations (registers) in which the peptide binds to the particular MHC molecule, and *δ*𝖲 is the difference in entropy between the bound and unbound states.

For the trans-allele model, two changes were introduced into the second term of equation (2). First, instead of residue-residue interaction, $\delta {\text{H}}^{\left(\text{2}\right)}\left({\text{a}}_{\text{ir}}^{\left(\text{k}\right)},{\text{b}}_{\text{j}}\right)$, with ${\text{a}}_{\text{ir}}^{\left(\text{k}\right)}$ on the peptide sequence and 𝖻_𝗃_ on the MHC binding pocket, we rather focus on residue-polymorphic group interaction, $\delta {\text{H}}^{\left(\text{2}\right)}\left({\text{a}}_{\text{ir}}^{\left(\text{k}\right)},{\text{g}}_{\text{jn}}\right)$, where 𝗀_𝗃𝗇_ is residue group number *n* of position *j* as defined in Section 2.1. Next, we introduce a binary operator 𝖳(𝗄, 𝗃, 𝗇) that equals 1 if the MHC molecule type, 𝖬^(𝖳(𝗄))^, corresponding to peptide 𝖯^(𝗄)^ contains polymorphic residue group *n* at the set of pre-determined positions of pocket *j*, and equals 0 otherwise. Hence, *δ*𝖤^(𝗄)^ is given by the following equation:

(3) $$\delta {\text{E}}^{\left(\text{k}\right)}=\underset{\text{first-order Hamiltonians}}{\underset{︸}{\underset{i=1}{\overset{\left|{\text{P}}^{\left(\text{k}\right)}\right|}{\sum }}\;+\delta H^{\left(1\right)}\left({\text{a}}_{i}\right)+\underset{i=1}{\overset{9}{\sum }}\;\delta H^{\left(1\right)}\left({\text{b}}_{i}\right)+\delta s}}+\overset{\text{per residue-group interactions}}{\overset{︷}{\underset{\text{second-order Hamiltonians}}{\underset{︸}{\underset{i=1}{\overset{\left|{\text{P}}^{\left(\text{k}\right)}\right|}{\sum }}\;\underset{j=1}{\overset{9}{\sum }}\;\underset{r=1}{\overset{\text{R}}{\sum }}\;\underset{n=1}{\overset{\text{G}\left(\text{j}\right)}{\sum }}\;\delta H^{\left(2\right)}\left({\text{a}}_{\mathrm{ir}}^{\left(k\right)},{\text{g}}_{\mathrm{jn}}\right)\text{T}\left(\text{k},\text{j},\text{n}\right)}}}},$$

where 𝖦(𝗃) is the number of polymorphic residue groups for binding pocket *j*. Column two of Table S2 in Supplementary Material shows 𝖦(𝗃), *j* = 1, 2, …, 9, for HLA-DR, HLA-DP, and HLA-DQ alleles.

Let Δ denote the model’s parameters. Using equations (1) and (3), we formulate, through the maximum likelihood approach, the following cost function:

(4) $$L\left(\text{P},\text{M}|\Delta \right)=\underset{\left\{\Delta \right\}}{\text{argmin}}\;\left(\underset{\text{k}=1}{\overset{\text{K}}{\sum }}\;{\text{G}}^{\text{k}}\left(\Delta^{\text{k}}\right)+\lambda P\left(\Delta \right)\right),$$

where ${\text{G}}^{\text{k}}\left(\Delta \right)$ is the empirical loss function given by the following equation:

(5) GkΔ=yk log (πkΔ)+1−yk log (1−πkΔ),

and 𝗒^𝗄^ ∈ {0, 1} is the experimental value; *y* = 1 for binding peptides and *y* = 0 for non-binding ones. λ𝒫(Δ) is a regularization term with the following form:

(6) $$\lambda P\left(\Delta \right)=\lambda {\left|\left|\Delta \right|\right|}_{1}=\lambda \underset{i=1}{\overset{d}{\sum }}\;\left|\Delta \right|,$$

where λ > 0 is a hyper-parameter and *d* is the dimension of parameter vector Δ, which varies depending on the type of MHC-II molecule. The *L*_1_ constraint penalty term 𝒫(Δ), also known as Lasso (38), has an important role in the model. As the model is defined on a large number of parameters (*d* = 2,321, 561, and 401 for HLA-DR, HLA-DP, and DQ molecules, respectively) a few parameters are expected to contribute to the binding affinity while the rest are expected to be noisy. Lasso has the capability to filter out the noisy parameters by inducing sparsity in the model, as it shrinks most of the parameter values to 0, and avoids data over-fitting. The hyper-parameter λ controls the degree of sparsity of the model; the larger the value of λ the more sparse the model. Equation (4) is a non-linear and non-smooth function; due to the *L*_1_ constraint. But it is a convex function and we solved it, after quadratic approximation, by means of an iterative, cyclic coordinate descent approach using a soft-thresholding operator. This learning function takes the form of a generalized linear model and the algorithm we used to solve it is both fast and efficient. Details of this optimization method are found in Friedman et al. (39) and are summarized in the supplementary material.

### 2.3 Binding Affinity Dataset

The model has been developed by using both quantitative peptide-binding data and MHC-II molecule sequences. We obtained a total of 51,023 peptide-binding data for 24 HLA-DR, 5 HLA-DP, and 6 HLA-DQ from the IEDB database (40). This is a well-curated dataset and was used to develop NetMHCIIpan (18), the state-of-the-art method. The binding affinities data were given in the form of log-transformed measurements of the IC_50_ (half maximum inhibition concentration) according to the formula 1 − log(*IC*_50_)/log(50,000) (16). We dichotomized these data using a moderate threshold of IC_50_ 500 nM (≡0.426 of log-transformed data). Peptides with *IC*_50_ less than or equal 500 nM (≥0.426 of log-transformed value) were considered as binders, and non-binders otherwise. This moderate threshold, which has been used in other previous methods including the state-of-the art method (20, 29, 30, 41), allows us to make direct comparisons.

Amino acid sequences for the MHC-II alleles used in this study were obtained from the EMBL-EBI online-database (42). Table 1 gives a summary of the peptide-binding dataset used to develop the method.

**Table 1:** Overview of the MHC-II peptide-binding data utilized in this study.

Allele name	HLA-index	# of Peptides	# of Binders	% of Binders
**HLA-DR molecules**				
*DRB*1*01:01	HLA00664	7,685	4,382	57.02
*DRB*1*03:01	HLA00671	2,505	649	25.91
*DRB*1*03:02	HLA00673	148	44	29.73
*DRB*1*04:01	HLA00685	3,116	1,039	33.31
*DRB*1*04:04	HLA00689	577	336	58.23
*DRB*1*04:05	HLA00690	1,582	627	39.63
*DRB*1*07:01	HLA00719	1,745	849	48.65
*DRB*1*08:02	HLA00724	1,520	431	28.36
*DRB*1*08:06	HLA00732	118	91	77.12
*DRB*1*08:13	HLA00739	1,370	455	33.21
*DRB*1*08:19	HLA00745	116	54	46.55
*DRB*1*09:01	HLA00749	1,520	621	40.86
*DRB*1*11:01	HLA00751	1,794	778	43.37
*DRB*1*12:01	HLA00789	117	81	69.23
*DRB*1*12:02	HLA00790	117	79	67.52
*DRB*1*13:02	HLA00798	1,580	493	31.20
*DRB*1*14:02	HLA00834	118	78	66.20
*DRB*1*14:04	HLA00836	30	16	53.33
*DRB*1*14:12	HLA00844	116	63	54.31
*DRB*1*15:01	HLA00865	1,769	709	40.08
*DRB*3*01:01	HLA00887	1,501	281	18.72
*DRB*3*03:01	HLA00902	160	70	43.75
*DRB*4*01:01	HLA00905	1,521	485	31.89
*DRB*5*01:01	HLA00915	3,106	1,280	41.21
**HLA-DP molecules**				
*DPA*1*01:03–*DPB*1*02:01	HLA00517	1,404	538	38.32
*DPA*1*01:03–*DPB*1*04:01	HLA00521	1,337	471	35.23
*DPA*1*02:01–*DPB*1*01:01	HLA00514	1,399	597	42.67
*DPA*1*02:01–*DPB*1*05:01	HLA00523	1,410	443	31.42
*DPA*1*03:01–*DPB*1*04:02	HLA00522	1,407	523	37.17
				
**HLA-DQ molecules**				
*DQA*1*01:01–*DQB*1*05:01	HLA00638	1,739	522	30.02
*DQA*1*01:02–*DQB*1*06:02	HLA00646	1,629	813	49.91
*DQA*1*03:01–*DQB*1*03:02	HLA00627	1,719	386	22.46
*DQA*1*04:01–*DQB*1*04:02	HLA00637	1,701	559	32.86
*DQA*1*05:01–*DQB*1*02:01	HLA00622	1,658	549	33.11
*DQA*1*05:01–*DQB*1*03:01	HLA00625	1,689	863	51.10
Total		51,023	20,255	39.70

*The first column gives the names of the 34 genes used to develop the method, distributed as 24, 5, and 6 for HLA-DR, HLA-DP, and HLA-DQ genes, respectively. The second column represents the index for each allele in the **EMBL-EBI** database (42). The third and fourth columns give the total number of peptide and the number of binder peptides, receptively, per allele. The last column shows the percentage of binder peptides. Binder peptides were identified using an *IC*_50_ binding cutoff of 500 nM, as in previous studies (2, 17, 18, 30). The last row presents the overall statistics for the last three columns*.

## 3 Results

This section presents prediction results of the model obtained from the dataset of three MHC-II allotypes as described in Section 2.3. We applied a fivefold cross validation analysis to the model and compared it against its intra-allelic version (Table S3 in Supplementary Material). We also examine its predictive performance on data which were previously unseen by the model.

### 3.1 Performance of the Trans-Allele Model

**Figure 1:**
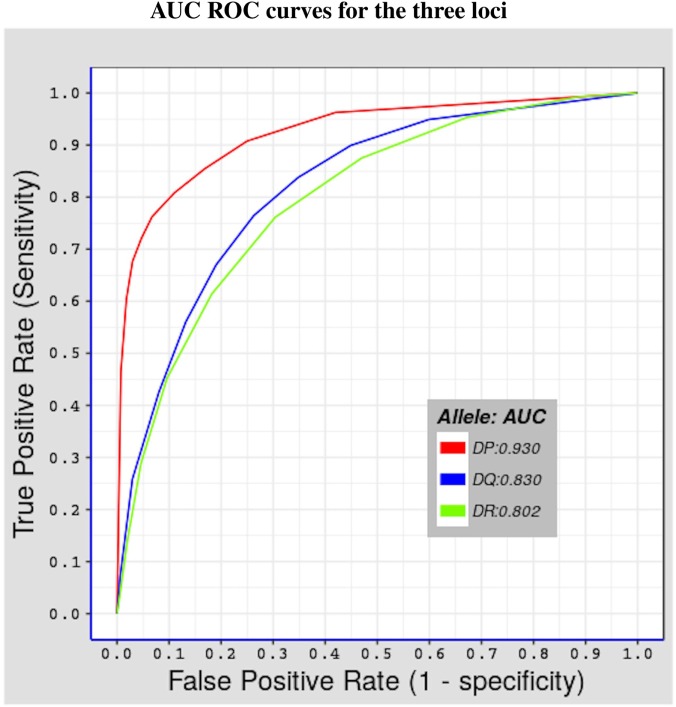
Shows fivefold cross validation results of the model using the benchmark dataset described in Section 2.3. Three ROC curves representing the three MHC-II loci covered in this study. The red curve for HLA-DP with AUC value = 0.930, the blue curve for HLA-DQ with AUC value = 0.830, and the green curve for HLA-DR with AUC = 0.802.

We tested the predictive performance of the model by using fivefold cross validation. The partitioning of the data used in fivefold cross validation was previously done by Andreatta et al. (29), by clustering together peptides in a way that minimizes over-estimation of predictive performance, using the technique described by Nielsen et al. (30). Figure 1 shows results of the test done using alleles belonging to the three MHC-II loci considered in this study. The performance was measured in terms of area under the curve (AUC) (43) values, which range between 0 and 1. The higher the AUC value the better the predictive performance of model. Values below 0.5 reflect a worse performance than a random test. The model has an excellent performance for HLA-DP molecules (average AUC value = 0.930), and a good predictive power for both HLA-DQ and HLA-DR molecules (average AUC values = 0.830 and 0.802, respectively). The surprisingly excellent performance for HLA-DP could be the result of both a higher structural similarity (see Section 3.3) and a higher number of peptides per allele for HLA-DP. Indeed, for all HLA-DP alleles, the number of available peptides exceeds the empirically required number of peptide-binding measurements (≈200 peptides (22)), but this is not the case for all HLA-DR alleles. HLA-DQ alleles have sufficient number of peptide measurements but these have a lower structural similarity compared with the corresponding peptides for HLA-DP alleles (see Section 3.3).

### 3.2 Comparing the Intra-Allele vs Trans-Allele Methods

**Figure 2:**
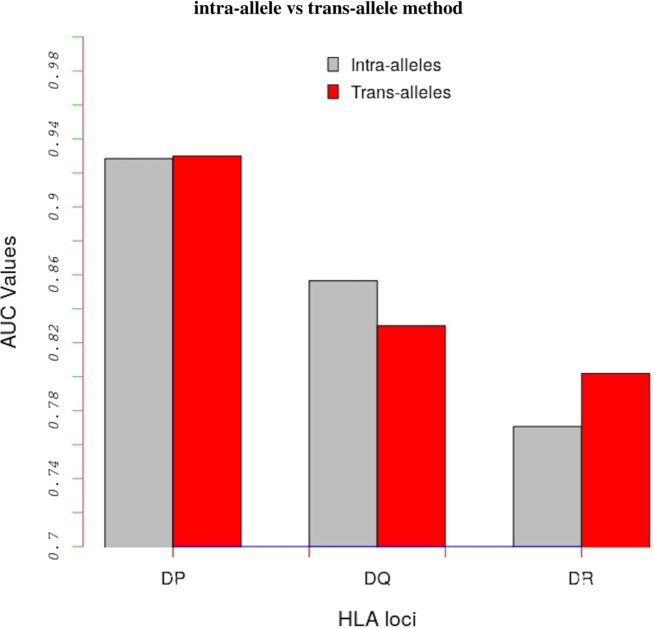
Comparing results between the intra-alleles (gray bars) and the trans-alleles (red bars) methods in terms of AUC values. These bars show that there is a significant increase in performance of the trans-allele method for HLA-DR molecules and decrease for HLA-DQ molecules compared with the intra-allele method. The difference in the HLA-DP loci is limited.

Table S3 in Supplementary Material shows AUC values obtained with the intra-allele and trans-allele versions of the model. For the intra-alleles version, the model was evaluated on peptide-binding data corresponding to an individual allele only. On average, the performance of the trans-allele model is comparable to that of the intra-allele model for HLA-DP (0.930 vs 0.928), it is worse for HLA-DQ (0.830 vs 0.857) and it is better for HLA-DR (0.780 vs 0.771) (Figure 2).

These results demonstrate two important observations. First, there is a common binding preference among MHC-II loci, which is the basis of all trans-allelic models, and that has been successfully captured by the definition of MHC-II polymorphic groups for HLA-DP loci, and to a lesser extent for HLA-DQ and HLA-DR. Second, the trans-allelic model is able to extrapolate similarities among the MHC-II allotypes and achieve good predictive performance. As a result, the overall performance of the trans-allelic model is comparable to that of intra-allele model, even though the former model is applied on a much diverse set of MHC-II sequences.

A decreased performance of the trans-allelic model when compared with the intra-allelic method for HLA-DQ molecules is consistent with results reported in NetMHCIIpan (18). Here we suggest that this is probably because of the limited structural information available for HLA-DQ alleles. In fact, because of this limited structural information there are only 17 polymorphic residue groups for all the 9 binding pockets defined for HLA-DQ alleles. By contrast, there are 25 and 115 polymorphic residue groups defined for HLA-DP and HLA-DR molecules, respectively.

Another reason for the reduction of the trans-allelic model’s performance for HLA-DQ alleles is that there is a large sequence diversity of MHC-II molecules belonging to this locus. We will examine the empirical support for this assertion in Section 3.3.

### 3.3 Prediction on a Novel Dataset

We examined the predictive power of the model on a blind dataset- i.e., a dataset which was not used in the training phase. More precisely, to make peptide-binding predictions for a particular allele, we train the model on an entirely different allele. The allele used for training was chosen based on its similarity to the focal allele as quantified using three different metrics: nearest-neighbor, Hamming distance, and Leave-One-Out (LOO) approach.

In the nearest-neighbor approach the distance between two MHC molecules is defined (17) as follows:

(7) $$d\left(A,B\right)=1-\frac{S\left(A,B\right)}{\sqrt{S\left(A,A\right)S\left(B,B\right)}}$$

in which *S*(*A, B*) is the score of the BLOSUM50 (44) metric between amino acid sequences of *A* and *B*. The BLOSUM50 metric measures genetic distance between two sequences by quantifying the likelihood that one amino acid will be substituted by another amino acid on evolutionary time scales. Hamming distance simply counts the different occurrences of corresponding amino acid residues between two sequences. In both nearest-neighbor and Hamming metrics, we train the model on peptide data belonging to the corresponding nearest allele to parameterize the model, and then we assess its accuracy in terms of AUC values calculated based on peptide data belonging to the focal allele using those parameters.

However, unlike the TEPITOPE and the series of NetMHCIIpan methods which defined nearest neighbor at pocket level, we derive both the nearest-neighbor metric and the Hamming distance at residue level. Our choice is based on the fact that accounting for the entire MHC-II sequence provides a broader allele coverage (2) and hence extend the model’s applicability. Computing sequence similarity at residue level is an intuitive and natural approach to perform comparative analysis of sequences rather than other artificial ways that may be more computationally efficient. We found that 71% (for HLA-DR), 60% (HLA-DP), and 67% (HLA-DQ) of alleles used for training were consistent between the residue-level and pocket-level approaches. These statistics indicate that, as mentioned before, most of MHC-II polymorphisms occur at the binding pockets.

**Figure 3:**
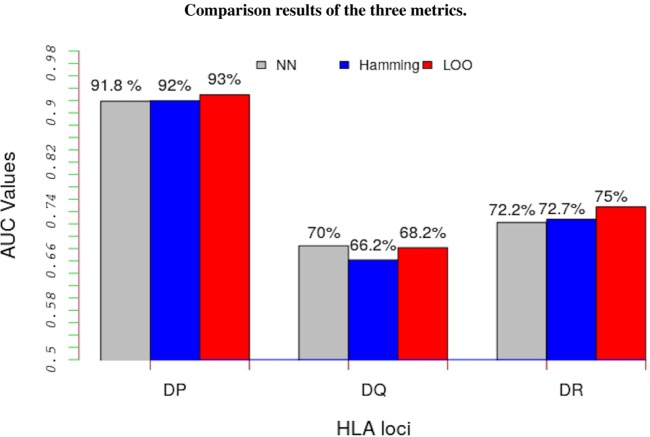
Average performance results of the model in terms of AUC values for the three metrics: NN approach (gray bars), Hamming metric (blue bars), and the LOO method (red bars). Except for HLA-DQ loci, the LOO approach significantly out performs the other two metrics. Such results indicate that this method performs better than a random test even for un-characterized MHC-II molecules.

The LOO approach involved partitioning data into two parts; the peptide-binding data not belonging to the allele under consideration are used to learn the model’s parameters and the remaining data, the peptide-binding data belonging to the focal allele, are used as test data. Figure 3 shows a comparison of results from these three approaches (details are in Table S4 in Supplementary Material). The results show that, regardless of the metric we used, the trans-allele method has a high predictive power for HLA-DP allele and a moderate predictive power for the other alleles.

**Figure 4:**
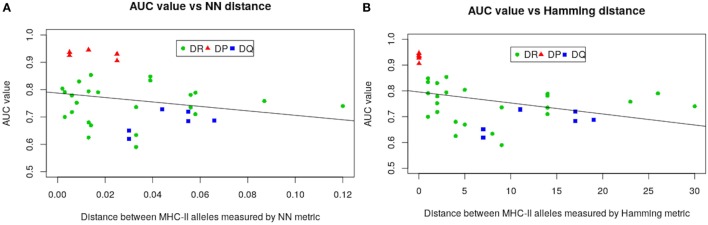
Regression analysis of AUC values from the LOO approach as function of: **(A)** nearest-neighbor and **(B)** Hamming distances. Negative slope lines in both graphs obtained by the least square fit method, with p-values 0.185 and 0.0.033 for both metrics, respectively. These lines and p-values associated with were produced using glm2 package in R (45).

The much higher predictive power for HLA-DP compared with the other alleles is likely due to the comparatively lower sequence diversity of HLA-DP alleles. To make this assertion more precise we carried out a regression analysis by defining the AUC values from LOO approach as functions of both NN and Hamming metric distances. Figure 4 gives results of our analysis. As seen in Figure 4, all HLA-DQ alleles fall below the least squares lines for both metrics (blue points). We also found that model performance for HLA-DP allele (red points) increases as the distance between alleles decreases. The authors of NetMHCIIpan also arrived at the same conclusion (18), but only for the NN metric.

### 3.4 Analysis of the Model’s Parameters

To determine the key factors that contribute to the binding affinities for the three MHC-II alleles considered in this study, we calculated the Hamiltonians corresponding to each amino acid residue and the 9 binding pockets of the MHC-II binding groove. These values were then averaged over only the polymorphic residue groups defined for each pocket containing the particular amino acid.

**Figure 5:**
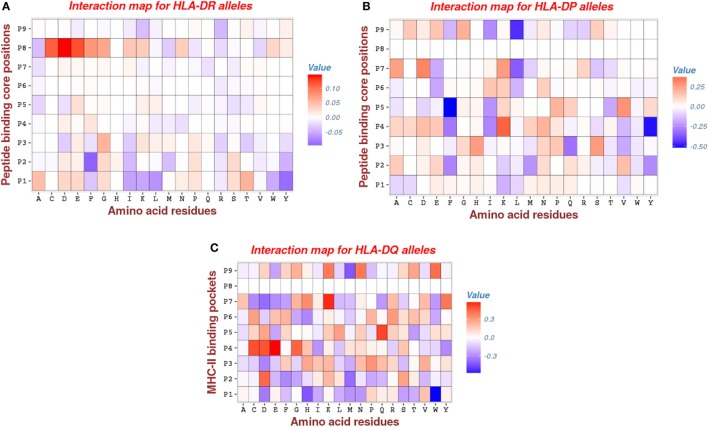
Interaction maps for: **(A)** HLA-DR, **(B)** HLA-DP, and **(C)** HLA-DQ molecules. The rows give the 9 anchor positions of MHC-II binding groove and the columns give the peptide residues. The red entries marking for repulsive interactions (positive energy), whereas the blue entries marking for attractive interactions (negative energy). Note that most of the entries are zeros (white color), an indication for the degree of the sparsity of the model.

Analysis of HLA-DR parameters revealed that pocket *P*1 has moderate attractive interactions with peptide (negative energies indicated by blue color in Figure 5), via hydrophobic **(I**, **L**, **W**, **Y)** side chains and, to lesser extent, via the aromatic **(F**, **W)** amino acids and a single hydrophilic residue **(K)**. Remarkably, previous studies (2, 46) arrived at a similar conclusion of a large tendency of position *P*1 toward interactions involving the hydrophobic side chains. The repulsive interactions (positive energies indicated by red color in Figure 5) of pocket *P*1 mostly occur with the hydrophilic side chains **(D**, **E**, **N**, **S**, **T)** and the aliphatic residue **(A)**. Generally, most of the primary anchor pockets (*P*1, *P*4, *P*6, *P*7, *P*9) confer attractive interactions, but the pocket *P*1 makes the largest contribution. This is consistent with results obtained using the MULTIRTA method (2). Among the secondary anchors, we found that pocket *P*2 has attractive interactions with aromatic **(F**, **Y)** and the hydrophobic **(I**, **M**, **Y)** side chains. The most repulsive interactions come from the pocket *P*8, which has a strong unfavorable interactions involving the side chains of residues **C**, **D**, **E**, **F**, **G**, **I**, **L**, **W**, and **Y** (see Figure 5A).

For HLA-DP, we found that pocket *P*9 has significantly attractive interactions involving the hydrophobic residue (**L**). This is consistent with the previous results of Ref. (47) (see Figure 5B). Also, we found that pockets *P*4 and *P*5 have important attractive interactions with peptide via hydrophobic **(Y)** and aromatic **(F)** side chains, respectively. The contribution of the pocket *P*4 is concordant with other studies such as (41), but the contribution of the pocket *P*5 was not reported in the study of Andreatta and Nielsen (47), which was specifically dedicated to HLA-DQ and HLA-DP alleles. Furthermore, we found that the other two pockets *P*1 and *P*6, which were reported as primary anchors in that study, have a moderate contribution to calculated bind energies (see Figure 5B).

The pattern of energetic contributions for HLA-DQ alleles is less ordered. There is no common pattern except the observation of significant attractive interaction of pocket *P*1 via the hydrophobic residue **(W)** and the repulsive interaction of pocket *P*4 via the side chains **C**, **E**, and **D** (see Figure 5C). This finding is in line with the observations of Morten et al. (47).

### 3.5 Discussion

Interactions between peptides and MHC-II molecules are central to the adaptive immune system. Precise prediction and knowledge of the physicochemical determinants that govern such interaction is useful in designing effective and affordable epitope-based vaccines, and in providing insights about the immune system’s mechanism as well as in understanding the pathogenesis of diseases. In this study, we have developed a trans-allelic model that can predict peptide interactions to the three human MHC-II loci. It can be readily applied to MHC-II molecules of other species provided that relative structural information are available. This method is based on biophysical ideas, an alternative to the dominant machine learning approaches.

The model presented here is, in addition to NetMHCIIpan, only the second trans-allelic method that allows comprehensive prediction analysis of peptide binding to all three human MHC-II loci. Most trans-allelic models for MHC-II peptides are restricted to HLA-DR and HLA-DP alleles. The TEPITOPEpan method (23), which is popular among immunologists and is the successor of a pioneer method in this field, is limited to HLA-DR alleles.

In this work we employed the definition of MHC polymorphic residue groups of the MULTIRTA method (2), which is more intuitive and inclusive than the MHC pseudo sequences of NetMHCIIpan (18), in developing our trans-allelic model. Utilizing new structural data for MHC-II complexes, which were not present when MULTIRTA was being developed, we extended that idea to cover all three human MHC-II loci. There exist similar exercises for capturing structural similarity among MHC molecules. The earlier works of Murthy and Stern (48) and Sinigaglia and Hammer (49) were mostly limited to HLA-DR molecules. But in a previous study (2), the “polymorphic residue groups” were shown to be useful for inferring the interaction energy. This physical way of capturing structural similarity among MHC molecules works well in our biophysical approach.

**Figure 6:**
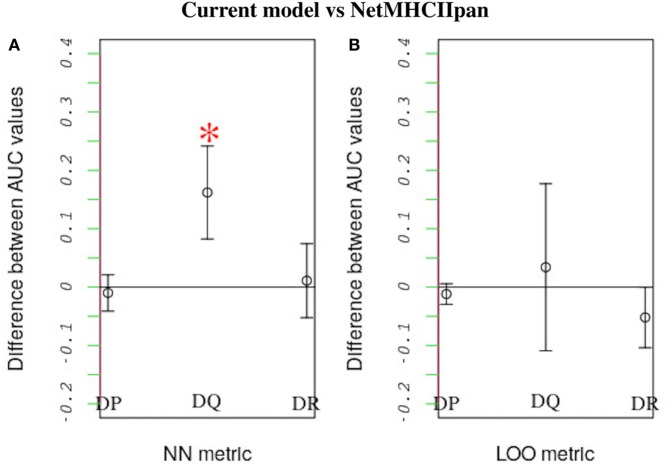
Performance comparison between our model and NetMHCIIpan. Each model was used to predict the probability of peptide binding to query alleles belonging to each of three HLA loci (i.e., HLA-DP, HLA-DQ, and HLA-DR) after training it using peptide-binding data for a different allele. The allele that was most similar to the query allele was used for training. As in previous work (18), similarity between HLA alleles was defined based on two metrics: nearest neighbor (NN) and leave-one-out (LOO). See the text for definitions of these metrics. For each query allele, we measured each model’s predictive performance (accounting for both sensitivity and specificity) by calculating an AUC value. The higher the AUC value the better the predictive performance. The plot shows the average difference between the AUC values for alleles belonging to the same locus obtained using our model vs. the corresponding values obtained using NetMHCIIpan, when similarity is defined based on either **(A)** the NN or **(B)** the LOO metric. Error bars denote SDs. Strikingly, our model performs better than NetMHCIIpan when predicting peptide binding to HLA-DQ using the NN metric (p-value = 0.015). For all other cases, both models have equivalent performance.

We compared how well our model predicts the MHC-II allele binding preferences of a novel peptide dataset vs. how well the state-of-the-art NetMHCIIpan method performs the same task. In this comparison we applied both our model and NetMHCIIpan to predict binding preferences for peptides known to either bind or not bind a reference allele after training both models using peptide-binding data for a second allele. For a given MHC-II locus, the second allele was the one that was most similar to the reference allele. Similarity was quantified based on either a leave-one-out approach or a nearest-neighbor approach (see Section 3.3). When using the nearest-neighbor approach, we found that our model performs significantly better than NetMHCIIpan in predicting peptide-binding preferences for HLA-DQ alleles (P-value = 0.015; Figure 6A). Furthermore, at the 95% confidence level, for all other cases, we found no significant difference between the performances of the two models (Figure 6).

These results are reassuring and indicate that our inverse-physics approach constitutes a promising complement to the widely used pattern-based approach to peptide–MHC-II binding predictions. The outstanding predictive accuracy of the NetMHCIIpan is not the result of its theoretical basis. Rather it derives from the use of sophisticated ensembles of neural networks, which are very powerful. However, our method has a distinguishing advantage over all the advanced machine learning models in that it is more physically meaningful. It is worth noting that our prediction results of peptide–MHC-II interaction were based on *in silico* analysis of real data. Additional, *in vivo* and *in vitro* investigations are needed to further validate the reported predictive performance.

## Data Availability Statement

The peptide dataset used to evaluate this method can be found in the [IEDB] (http://tools.iedb.org/main/datasets/), and the MHC-II sequences data also can be found in the [EMBL-EBI] (ftp://ftp.ebi.ac.uk/pub/databases/ipd/imgt/hla/fasta/DRB_prot.fasta).

## Author Contributions

All authors contributed equally to this work.

## Conflict of Interest Statement

The authors declare that the research was conducted in the absence of any commercial or financial relationships that could be construed as a potential conflict of interest.
