# Supplementary material for: Trans-Allelic Model for Prediction of Peptide:MHC-II Interactions
Source: Front Immunol. 2018 Jun 20;9:1410. doi: 10.3389/fimmu.2018.01410 (PMC6026802; doi:10.3389/fimmu.2018.01410)
Supplement: Supplementary file 1 [file Table_1.PDF]

Supplementary Material: Trans-Allelic Model for Prediction of Peptide:MHC-II Interactions

1 SUMMARY OF THE OPTIMIZATION ALGORITHM

Let x_k be the input vector that associated with a given pair of peptide $\mathsf{P}^{(\mathsf{k})}$ and MHC-II molecule $\mathsf{M}^{(\mathsf{T}(\mathsf{k}))}$, which can be extracted from 3-D structural information over all possible registers, and let $y_k \in \{0, 1\}$ be the binary experimental value. For sake of an easy notation, let θ denotes for the model parameter (instead of Δ in the main text) representing the Hamiltonians.

The empirical loss function of **Eq5** in the main text will take the following form:

$$\mathsf{G}_\mathsf{k}(\theta) = \mathsf{y}_\mathsf{k} \log(\pi_\mathsf{k}(\theta)) + (1 - \mathsf{y}_\mathsf{k}) \log(1 - \pi_\mathsf{k}(\theta)), \tag{S1}$$

where

$$\pi_\mathsf{k}(\theta) = \frac{1}{1 + \exp(x_k^T \theta)}.$$

Equation S1 could be simply written as follows:

$$\mathsf{G}_\mathsf{k}(\theta) = (1 - y_k) x_k^T \theta - \log(1 + \exp(x_k^T \theta)). \tag{S2}$$

Then the learning function of **Eq4** in the main text is:

$$\mathcal{L}(\theta) = \underset{\{\theta\}}{\operatorname{argmin}} \left[-G(\theta) + \lambda ||\theta||_1\right] = \sum_{k=1}^{N} \Big((y_k - 1) x_k^T \theta + \log(1 + \exp(x_k^T \theta)) \Big) + \lambda \sum_{j=1}^{d} |\theta_j|, \tag{S3}$$

where N is total number of the given data points and d is the dimension of the parameter vector.

In order to get a closed formula for the update rule, we need to put the first sum of equation S3, $G(\theta)$, in a quadratic form using Taylor expansion around a given point θ_0.

$$G_Q(\theta) \approx G(\theta_0) + (\theta - \theta_0)^T \frac{\partial G(\theta_0)}{\partial \theta} + \frac{1}{2} (\theta - \theta_0)^T \frac{\partial^2 G(\theta_0)}{\partial \theta^2} (\theta - \theta_0) + C^2(\theta_0), \tag{S4}$$

such that $C^2(\theta_0)$ is the truncated error of the second order in vicinity of the current point θ_0. The first and second derivatives are:

$$\frac{\partial G(\theta)}{\partial \theta} = \sum_{k=1}^{N} x_k^T \,(y_k - \bar{\pi}_k)$$

$$\frac{\partial^2 G(\theta)}{\partial \theta^2} = \sum_{k=1}^{N} x_k^T \bar{\pi}_k (1 - \bar{\pi}_k)) x_k$$

where $\bar{\pi}_k = \pi_k(\theta_0)$ is the value of probability evaluated at point θ_0. Therefore, the quadratic approximation is:

$$G_Q(\theta) \approx \sum_{k=1}^{N} \left[x_k^T(\theta-\theta_0)\bar{\pi}_k(1-\bar{\pi}_k))x_k^T(\theta-\theta_0) \right] + \sum_{k=1}^{N} \left[x_k^T(\theta-\theta_0)(y_k-\bar{\pi}_k \Big) \right] + G(\theta_0) + C^2(\theta_0). \tag{S5}$$

Let $w_k = \bar{\pi}_k(1-\bar{\pi}_k)$, which is called re-weighting term, and re-write the above equation as follows:

$$G_Q(\theta) \approx \frac{1}{2}\sum_{k=1}^{N} w_k \left[\Big(x_k^T(\theta-\theta_0) \Big)^2 + 2\frac{x_k^T(\theta-\theta_0)(y_k\bar{\pi}_k)}{w_k} \right] + G(\theta_0) + C^2(\theta_0). \tag{S6}$$

And even more simplified

$$G_Q(\theta) \approx \frac{1}{2}\sum_{k=1}^{N} w_k \left[x_k^T(\theta-\theta_0) + \frac{y_k-\bar{\pi}_k}{w_k} \right]^2 + C(\theta_0), \tag{S7}$$

where $C(\theta_0)$ is an augmented constant, i.e. $C(\theta_0) = -\frac{1}{2}\sum_{k=1}^{N} \frac{y_k-\bar{\pi}_k}{w_k} + G(\theta_0) + C^2(\theta_0)$. Define the constant $z_k = x_k^T\theta_0 + \frac{\bar{pi}_k - y_k}{w_k}$, and hence

$$G_Q(\theta) \approx \frac{1}{2}\sum_{k=1}^{N} w_k \left[z_k - x_k^T\theta \right]^2 + C(\theta_0). \tag{S8}$$

By plugging equation S8 into equation S3 we get the a quadratic form of the optimization function

$$G_Q(\theta) \approx \frac{1}{2}\sum_{k=1}^{N} w_k \left[z_k - x_k^T\theta \right]^2 + \lambda\sum_{j=1}^{d} |\theta_j| + C(\theta_0). \tag{S9}$$

The above equation is convex but not differentiable; due to the L_1 component, and we solved via iterative cyclic coordinate descent algorithm.

$$\frac{\partial G_Q(\theta)}{\partial \theta_j} = 0 \Rightarrow -\sum_{k=1}^{N} \left[z_k - x_k^T\theta \right] x_{kj} + \lambda\frac{\partial|\theta_j|}{\partial\theta} = 0$$

$$\Rightarrow \sum_{k=1}^{N} w_k(x_{kj})^2 - \sum_{k=1}^{N} w_k \left[z_k - \sum_{r=1;r\neq j}^{d} x_{kr}\theta_r \right] x_{kj} + \lambda\frac{\partial|\theta_j|}{\partial\theta} = 0.$$

For simplicity let $a_j = \sum_{k=1}^{N} w_k(x_{kj})^2$ and $b_j = \sum_{k=1}^{N} w_k \left[z_k - \sum_{r=1;r\neq j}^{d} x_{kr}\theta_r \right] x_{kj}$. Then

$$a_j\theta_j - b_j + \lambda\frac{\partial|\theta_j|}{\partial\theta} = 0.$$

Using idea of sub-differential

$$\begin{cases} a_j\theta_j - b_j + \lambda, & \text{if } \theta_j > 1 \\ a_j\theta_j - b_j - \lambda, & \text{if } \theta_j < -1 \\ 0, & \text{otherwise} \end{cases}$$

As a_j is absolutely non-negative, therefore the sign of θ_j depends on $\ni_j$ and λ. Then

$$\theta_j = \begin{cases} \frac{b_j-\lambda}{a_j}, \text{if } bj > 0 \text{ and } \lambda < |bj| \\ \frac{b_j+\lambda}{a_j}, \text{if } b_j < 0 \text{ and } \lambda < |b_j| \\ 0, \quad \text{if } \lambda \leq |b_j|. \end{cases} \tag{S10}$$

Equation S10 known as soft-threshold operator.

The complete procedures of algorithm are following:

1. Initialize values for $\lambda, \lambda_{\text{min}}, \epsilon$, and θ_0.
2. For every j in the parameter vector
   a. Use the current value of θ to compute a_j and b_j for the jth component.
   b. Use a_j and b_j to calculate new value for θ_j in equation S10.
   c. Repeat (a) and (b) until convergent.
3. Decrease λ by $\lambda = \epsilon\lambda_{\text{current}}$
4. While $\lambda > \lambda_{\text{min}}$ repeat

Remark: The re-weighting term $w_k = \bar{\pi}_k(1 - \bar{\pi}_k)$ might go to zero and leads to a divergent results. In such cases we used its upper bound, i.e $w_k = 0.25$ (see Friedman et al. (2010)).

2 LIST OF TABLES

Table S1: **Peptide:MHC-II complex structures**

**PDB Index**	**Allele**	**Peptide sequence**
1AQD	$DRB1$*01 : 01	VGSDWRFLRGYHQYA
1PYW	$DRB1$*01 : 01	XFVKQNAAALX
1KLG	$DRB1$*01 : 01	GELIGILNAAKVPAD
1KLU	$DRB1$*01 : 01	GELIGTLNAAKVPAD
2FSE	$DRB1$*01 : 01	AGFKGEQGPKGEPG
1SJH	$DRB1$*01 : 01	PEVIPMFSALSEG
1SJE	$DRB1$*01 : 01	PEVIPMFSALSEGATP
1T5W	$DRB1$*01 : 01	AAYSDQATPLLLSPR
1T5X	$DRB1$*01 : 01	AAYSDQATPLLLSPR
2IAN	$DRB1$*01 : 01	GELIGTLNAAKVPAD
2IAM	$DRB1$*01 : 01	GELIGILNAAKVPAD
2IPK	$DRB1$*01 : 01	XPKWVKQNTLKLAT
1FYT	$DRB1$*01 : 01	PKYVKQNTLKLAT

Continued on next page

Table S1 – continued from previous page

1R5I	$DRB1^{*}01:01$	PKYVKQNTLKLAT
1HXY	$DRB^{*}10101$	PKYVKQNTLKLAT
1JWM	$DRB1^{*}01:01$	PKYVKQNTLKLAT
1JWS	$DRB1^{*}01:01$	PKYVKQNTLKLAT
1JWU	$DRB1^{*}01:01$	PKYVKQNTLKLAT
1LO5	$DRB1^{*}01:01$	PKYVKQNTLKLAT
2ICW	$DRB1^{*}01:01$	PKYVKQNTLKLAT
2OJE	$DRB1^{*}01:01$	PKYVKQNTLKLAT
2G9H	$DRB1^{*}01:01$	PKYVKQNTLKLAT
1A6A	$DRB1^{*}03:01$	PVSKMRMATPLLMQA
1J8H	$DRB1^{*}04:01$	PKYVKQNTLKLAT
2SEB	$DRB1^{*}04:01$	AYMRADAAAGGA
1BX2	$DRB1^{*}15:01$	ENPVVHFFKNIVTPR
1YMM	$DRB1^{*}15:01$	ENPVVHFFKNIVTPRGGSGGGGG
1FV1	$DRB5^{*}01:01$	NPVVHFFKNIVTPRTPPPSQ
1H15	$DRB5^{*}01:01$	GGVYHFVKKHVHES
1ZGL	$DRB5^{*}01:01$	VHFFKNIVTPRTPGG
4E41	$DRB1^{*}01:01$	GELIGILNAAKVPAD
1DLH	$DRB1^{*}01:01$	PKYVKQNTLKLAT
1KG0	$DRB1^{*}01:01$	PKYVKQNTLKLAT
3L6F	$DRB1^{*}01:01$	APPAYEKLSAEQSPP
3PDO	$DRB1^{*}01:01$	KPVSKMRMATPLLMQALPM
3PGD	$DRB1^{*}01:01$	KMRMATPLLMQALPM
3S4S	$DRB1^{*}01:01$	PKYVKQNTLKLAT
3S5L	$DRB1^{*}01:01$	PKYVKQNTLKLAT
1HQR	$DRB5^{*}01:01$	VHFFKNIVTPRTP
3LQZ	$DPB1^{*}02:01$	RKFHYLPFLPSTGGS
1UVQ	$DQB1^{*}06:02$	MNLPSTKVSWAAVGGGGSLV
1JK8	$DQB1^{*}03:02$	LVEALYLVCGERGG
1S9V	$DQB1^{*}02:01$	LQPFPQPELPY

Table S1: Presents peptides MHC-II structural complexes collected from from references Andreatta et al. (2015); Zhang et al. (2012); Zhao Li and Guo (2016). The first column gives the index of the protein databank, the second column presents the allele name of the beta chain, and the third column shows the peptides corresponding to the allele.

Table S2: **MHC-II polymorphic residue groups for HLA-DR genes.**

Binding Pocket	# of groups	polymorphic residue groups
**HLA-DRB molecules**		
P_1	4	{82N, 85A, 86V, 89F}, {82N, 85V, 86G, 89F}, {82N, 85V, 86V, 89F }, {82Y, 85G, 86E, 89T}
P_2	5	{77T, 78Y, 81H, 82N}, {77N, 78Y, 81H, 82N}, {77T, 78V, 81H, 82N}, {77Y, 78C, 81N, 82Y}, {77T, 78Y, 81Y, 82N}
P_3	8	{ 74A, 78Y}, {74E, 78V}, {74E, 78Y}, {74L, 78Y}, {74Q, 78V}, {74Q, 78Y}, {74R, 78Y}, {74V, 78C}
P_4	20	{ 11L, 13F, 14E, 26L, 28E, 70Q, 71R, 74A, 78Y}, { 11S, 13S, 14E, 26Y, 28D, 70Q, 71K, 74R, 78Y}, { 11S, 13S, 14E, 26F, 28E, 70Q, 71K, 74R, 78Y}, { 11V, 13H, 14E, 26F, 28D, 70Q, 71K, 74A, 78Y}, { 11V, 13H, 14E, 26F, 28D, 70Q, 71R, 74A, 78Y}, { 11G, 13Y, 14K, 26F, 28E, 70D, 71R, 74Q, 78V}, { 11S, 13G, 14E, 26F, 28D, 70D, 71R, 74L, 78Y}, { 11S, 13G, 14E, 26F, 28D, 70R, 71R, 74V, 78C}, { 11D, 13E, 14F, 26Y, 28H, 70R, 71R, 74E, 78V}, { 11S, 13S, 14E, 26F, 28D, 70D, 71R, 74A, 78Y}, { 11S, 13G, 14E, 26L, 28E, 70D, 71R, 74A, 78Y}, { 11S, 13S, 14E, 26F, 28D, 70D, 71E, 74A, 78Y}, { 11S, 13S, 14E, 26F, 28E, 70Q, 71R, 74A, 78Y}, { 11S, 13G, 14E, 26F, 28D, 70R, 71R, 74E, 78Y}, { 11S, 13S, 14E, 26F, 28E, 70D, 71R, 74L, 78Y}, { 11P, 13R, 14E, 26F, 28D, 70Q, 71A, 74A, 78Y}, { 11R, 13S, 14E, 26Y, 28D, 70Q, 71K, 74R, 78Y}, { 11L, 13S, 14E, 26F, 28E, 70Q, 71K, 74Q, 78Y}, { 11A, 13C, 14E, 26N, 28I, 70R, 71R, 74E, 78Y}, { 11D, 13Y, 14E, 26F, 28H, 70D, 71R, 74A, 78Y}
P_5	20	{11L, 13F, 28E, 30C, 70Q, 71R, 74A}, {11S, 13S, 28D, 30Y, 70Q, 71K, 74R}, {11S, 13S, 28E, 30Y, 70Q, 71K, 74R}, {11V, 13H, 28F, 30Y, 70Q, 71K, 74A}, {11V, 13H, 28D, 30Y, 70Q, 71R, 74A}, {11G, 13Y, 28E, 30L, 70D, 71R, 74Q}, {11S, 13G, 28D, 30Y, 70D, 71R, 74L}, {11S, 13G, 28D, 30Y, 70R, 71R, 74V}, {11D, 13F, 28H, 30G, 70R, 71R, 74E}, {11S, 13S, 28D, 30Y, 70D, 71R, 74A}, {11S, 13G, 28E, 30H, 70D, 71R, 74A}, {11S, 13S, 28D, 30Y, 70D, 71E, 74A}, {11S, 13S, 28E, 30Y, 70Q, 71R, 74A}, {11S, 13G, 28D, 30Y, 70R, 71R, 74E}, {11S, 13S, 28E, 30Y, 70D, 71R, 74L}, {11P, 13R, 28D, 30Y, 70Q, 71A, 74A}, {11R, 13S, 28D, 30Y, 70Q, 71K, 74R}, {11L, 13S, 28E, 30Y, 70Q, 71K, 74Q}, {11A, 13C, 28I, 30Y, 70R, 71R, 74E}, {11D, 13Y, 28H, 30D, 70D, 71R, 74A}

Continued on next page

Table S2 – continued from previous page

P_6	20	{9W, 11L, 13F, 28E, 30C, 70Q, 71R, 74A }, {9E, 11S, 13S, 28D, 30Y, 70Q, 71K, 74R }, {9E, 11S, 13S, 28E, 30Y, 70Q, 71K, 74R }, {9E, 11V, 13H, 28D, 30Y, 70Q, 71K, 74A }, {9E, 11V, 13H, 28D, 30Y, 70Q, 71R, 74A }, {9W, 11G, 13Y, 28E, 30L, 70D, 71R, 74Q }, {9E, 11S, 13G, 28D, 30Y, 70D, 71R, 74L }, {9E, 11S, 13G, 28D, 30Y, 70R, 71R, 74V }, {9K, 11D, 13F, 28H, 30G, 70R, 71R, 74E }, {9E, 11S, 13S, 28D, 30Y, 70D, 71R, 74A }, {9E, 11S, 13G, 28E, 30H, 70D, 71R, 74A }, {9E, 11S, 13S, 28D, 30Y, 70D, 71E, 74A },{9E, 11S, 13S, 28E, 30Y, 70Q, 71R, 74A }, {9E, 11S, 13G, 28D, 30Y, 70R, 71R, 74E }, {9E, 11S, 13S, 28E, 30Y, 70D, 71R, 74L }, {9W, 11P, 13R, 28D, 30Y, 70Q, 71A, 74A }, {9E, 11R, 13S, 28D, 30Y, 70Q, 71K, 74R }, {9E, 11L, 13S, 28E, 30Y, 70Q, 71K, 74Q }, {9E, 11A, 13C, 28I, 30Y, 70R, 71R, 74E }, {9Q, 11D, 13Y, 28H, 30D, 70D, 71R, 74A }
P_7	21	{11L, 28E, 30C, 47Y, 61W, 67L, 70Q, 71R}, {11S, 28D, 30Y, 47F, 61W, 67L, 70Q, 71K}, {11S, 28E, 30Y, 47Y, 61W, 67L, 70Q, 71K}, {11V, 28D, 30Y, 47Y, 61W, 67L, 70Q, 71K}, {11V, 28D, 30Y, 47Y, 61W, 67L, 70Q, 71R}, {11G, 28E, 30L, 47Y, 61W, 67I, 70D, 71R}, {11S, 28D, 30Y, 47Y, 61W, 67F, 70D, 71R}, {11S, 28D, 30Y, 47Y, 61W, 67L, 70D, 71R}, {11S, 28D, 30Y, 47Y, 61W, 67L, 70R, 71R}, {11D, 28H, 30G, 47Y, 61W, 67F, 70R, 71R}, {11S, 28D, 30Y, 47F, 61W, 67F, 7D0, 71R}, {11S, 28E, 30H, 47F, 61W, 67I, 70D, 71R}, {11S, 28E, 30H, 47F, 61W, 67F, 70D, 71R}, {11S, 28D, 30Y, 47F, 61W, 67I, 70D, 71E}, {11S, 28E, 30Y, 47Y, 61W, 67L, 70Q, 71R}, {11S, 28E, 30Y, 47Y, 61W, 67L, 70D, 71R}, {11P, 28D, 30Y, 47F, 61W, 67I, 70Q, 71A}, {11R, 28D, 30Y, 47Y, 61W, 67L, 70Q, 71K}, {11L, 28E, 30Y, 47Y, 61W, 67L, 70Q, 71K}, {11A, 28I, 30Y, 47Y, 61W, 67L, 70R, 71R}, {11D, 28Y, 30D, 47Y, 61W, 67F, 70D, 71R}
P_8	3	{60H, 61W}, {60S, 61W}, {60Y, 61W}
P_9	14	{9W, 30C, 37S, 38V, 57D, 60Y, 61W}, {9E, 30N, 37Y, 38V, 57D, 60Y, 61W}, {9E, 30Y, 37Y, 38V, 57D, 60Y, 61W}, {9E, 30Y, 37Y, 38V, 57S, 60Y, 61W}, {9W, 30L, 37F, 38V, 57V, 60S, 61W}, {9E, 30Y, 37Y, 38V, 57I, 60Y, 61W}, {9K, 30N, 37g, 38V, 57V, 60S, 61W}, {9E, 30H, 37L, 38L, 57V, 60S, 61W}, {9E, 30Y, 37F, 38V, 57A, 60H, 61W}, {9W, 30Y, 37S, 38V, 57D, 60Y, 61W}, {9E, 30Y, 37F, 38L, 57V, 60S, 61W}, {9E, 30Y, 37F, 38V, 57V, 60S, 61W}, {9E, 30Y, 37Y, 38A, 57D, 60Y, 61W}, {9Q, 30D, 37D, 38L, 57D, 60Y, 61W}
**HLA-DP molecules**		
P_1	2	{86G, 89M}, {86D, 89V}
P_2	2	{78M}, {78V}
P_3	2	{78M}, {78V}
P_4	4	{71E}, {71K}, {71V}, {78V}
P_5	2	{71E}, {71K}
P_6	4	{9F}, {9Y},{71E}, {71K}
P_7	2	{71E}, {71K}
P_8	-	**NA**
P_9	7	{9F }, {37F }, {38V}, {57D}, {9Y, 37Y}, {37L, 57E}, {38A, 57A }

Continued on next page

Table S2 – continued from previous page

**HLA-DQ molecules**		
P_1	2	{86T, 89Q}, {86G, 89Q}
P_2	1	{78H}
P_3	1	{78H}
P_4	3	{71A, 78H}, {71E, 78H}, {71S, 78H}
P_5	3	{71A}, {71E}, {71S}
P_6	3	{9K, 71A}, {9K, 71E}, {9K, 71S}
P_7	3	{71A}, {71E}, {71S}
P_8	-	**NA**
P_9	1	{9K, 37F, 38D, 57Y }

Table S2: Shows the polymorphic residue groups for 24 HLA-DRB, 5 HLA-DP and 6 HLA-DQ molecules for each of 9 binding pockets. The first column gives the nine binding pockets, the second column gives the number of polymorphic residue groups per each binding pocket, and the last column gives the polymorphic groups shown by both the residue position number and amino acid type.

Table S3: **Results of five-fold cross results for intra-allele vs trans-allele in terms of AUC values**

**Allele Name**	**AUC**	
	**Intra-Allele training**	**Trans-Alleles training**
**HLA-DP molecules**		
$DPA1^{*}01:03-DPB1^{*}02:01$	0.933	0.933
$DPA1^{*}01:03-DPB1^{*}04:01$	0.935	0.939
$DPA1^{*}02:01-DPB1^{*}01:01$	0.921	0.922
$DPA1^{*}02:01-DPB1^{*}05:01$	0.925	0.926
$DPA1^{*}03:01-DPB1^{*}04:02$	0.928	0.927
**Overall**	**0.928**	**0.929**
**p-value**	**0.594**	**0.031**
**HLA-DQ molecules**		
$DQA1^{*}01:01-DQB1^{*}05:01$	0.864	0.833
$DQA1^{*}01:02-DQB1^{*}06:02$	0.830	0.815
$DQA1^{*}03:01-DQB1^{*}03:02$	0.791	0.728

Continued on next page

Table S3 – continued from previous page

$DQA1*04:01-DQB1*04:02$	0.883	0.873
$DQA1*05:01-DQB1*02:01$	0.887	0.871
$DQA1*05:01-DQB1*03:01$	0.884	0.799
**Average**	**0.857**	**0.820**
**p-value**	**0.4219**	**0.047**
**HLA-DRB molecules**		
$DRB1*01:01$	0.785	0.786
$DRB1*03:01$	0.747	0.725
$DRB1*03:02$	0.554	0.657
$DRB1*04:01$	0.774	0.756
$DRB1*04:04$	0.712	0.744
$DRB1*04:05$	0.781	0.794
$DRB1*07:01$	0.809	0.825
$DRB1*08:02$	0.725	0.716
$DRB1*08:06$	0.852	0.880
$DRB1*08:13$	0.821	0.837
$DRB1*08:19$	0.798	0.790
$DRB1*09:01$	0.753	0.757
$DRB1*11:01$	0.804	0.833
$DRB1*12:01$	0.818	0.824
$DRB1*12:02$	0.779	0.848
$DRB1*13:02$	0.761	0.718
$DRB1*14:02$	0.792	0.818
$DRB1*14:04$	0.674	0.674
$DRB1*14:12$	0.884	0.878
$DRB1*15:01$	0.783	0.784
$DRB3*01:01$	0.710	0.674
$DRB3*03:01$	0.769	0.765
$DRB4*01:01$	0.818	0.802
$DRB5*01:01$	0.793	0.820
**Average**	**0.771**	**0.780**
**p-value**	**0.169**	**0.329**

Table S3: The first column gives the allele name and other two columns provide performance measurements of five-fold cross validation in term area under the ROC curve of-(AUC) Fawcett (2006) values for intra-allele, by applying our previous method Degoot et al. (2017), and trans-allelic versions, respectively. Average and p-value (using Wilcoxon signed rank test Hollander and Wolfe (1999)) statics were also provided for all three MHC-II allotypes.

Table S4: Comparison results between nearest neighbourhood, Hamming distance and LOO method

Query allele	NN allele	NN distance	NN AUC	H allele	H distance	H AUC	LOO AUC
**HLA-DRB molecules**							
$DRB1^{*}01:01$	$DRB1^{*}15:01$	0.056	0.703	$DRB1^{*}15:01$	14	0.703	0.735
$DRB1^{*}03:01$	$DRB1^{*}03:02$	0.013	0.553	$DRB1^{*}03:02$	4	0.553	0.680
$DRB1^{*}03:02$	$DRB1^{*}03:01$	0.013	0.618	$DRB1^{*}14:02$	4	0.710	0.625
$DRB1^{*}04:01$	$DRB1^{*}04:05$	0.006	0.705	$DRB1^{*}04:05$	2	0.705	0.718
$DRB1^{*}04:04$	$DRB1^{*}04:01$	0.008	0.724	$DRB1^{*}04:05$	2	0.765	0.752
$DRB1^{*}04:05$	$DRB1^{*}04:01$	0.006	0.758	$DRB1^{*}04:04$	2	0.722	0.779
$DRB1^{*}07:01$	$DRB1^{*}09:01$	0.058	0.748	$DRB1^{*}09:01$	14	0.748	0.789
$DRB1^{*}08:02$	$DRB1^{*}08:13$	0.003	0.702	$DRB1^{*}08:13$	1	0.702	0.700
$DRB1^{*}08:06$	$DRB1^{*}08:02$	0.009	0.739	$DRB1^{*}08:02$	2	0.739	0.830
$DRB1^{*}08:13$	$DRB1^{*}08:02$	0.003	0.714	$DRB1^{*}08:02$	1	0.714	0.791
$DRB1^{*}08:19$	$DRB1^{*}08:13$	0.017	0.819	$DRB1^{*}08:13$	26	0.819	0.790
$DRB1^{*}09:01$	$DRB1^{*}07:01$	0.058	0.696	$DRB1^{*}07:01$	14	0.696	0.710
$DRB1^{*}11:01$	$DRB1^{*}13:02$	0.002	0.649	$DRB1^{*}13:02$	5	0.649	0.804
$DRB1^{*}12:01$	$DRB1^{*}12:02$	0.039	0.951	$DRB1^{*}12:02$	1	0.951	0.834
$DRB1^{*}12:02$	$DRB1^{*}12:01$	0.039	0.954	$DRB1^{*}12:01$	1	0.954	0.848
$DRB1^{*}13:02$	$DRB1^{*}14:02$	0.014	0.593	$DRB1^{*}11:01$	5	0.633	0.669
$DRB1^{*}14:02$	$DRB1^{*}03:02$	0.013	0.675	$DRB1^{*}14:12$	3	0.754	0.794
$DRB1^{*}14:04$	$DRB1^{*}08:06$	0.033	0.790	$DRB1^{*}14:12$	8	0.696	0.634
$DRB1^{*}14:12$	$DRB1^{*}14:02$	0.014	0.777	$DRB1^{*}14:02$	3	0.777	0.854

Continued on next page

Table S4 – continued from previous page

$DRB1$*15 : 01	$DRB1$*01 : 01	0.056	0.766	$DRB1$*01 : 01	14	0.766	0.781
$DRB3$*01 : 01	$DRB3$*03 : 01	0.033	0.579	$DRB3$*03 : 01	9	0.579	0.590
$DRB3$*03 : 01	$DRB3$*01 : 01	0.033	0.681	$DRB3$*01 : 01	9	0.681	0.736
$DRB4$*01 : 01	$DRB1$*04 : 04	0.120	0.698	$DRB1$*04 : 04	30	0.698	0.740
$DRB5$*01 : 01	$DRB1$*01 : 01	0.087	0.743	$DRB1$*01 : 01	23	0.743	0.758
**Average**			0.722			0.727	0.748
**p-value**			0.063			0.060	0.0612
**HLA-DP molecules**							
$DPA1$*02 : 01 – $DPB1$*01 : 01	$DPA1$*02 : 01 – $DPB1$*05 : 01	0.025	0.888	$DPA1$*02 : 01 – $DPB1$*05 : 01	8	0.888	0.906
$DPA1$*01 : 03 – $DPB1$*02 : 01	$DPA1$*03 : 01 – $DPB1$*04 : 02	0.005	0.929	$DPA1$*03 : 01 – $DPB1$*04 : 02	2	0.929	0.937
$DPA1$*01 : 03 – $DPB1$*04 : 01	$DPA1$*03 : 01 – $DPB1$*04 : 02	0.013	0.936	$DPA1$*03 : 01 – $DPB1$*01 : 01	4	0.941	0.946
$DPA1$*03 : 01 – $DPB1$*04 : 02	$DPA1$*01 : 03 – $DPB1$*02 : 01	0.005	0.910	$DPA1$*01 : 03 – $DPB1$*02 : 01	2	0.910	0.926
$DPA1$*02 : 01 – $DPB1$*05 : 01	$DPA1$*02 : 01 – $DPB1$*01 : 01	0.025	0.931	$DPA1$*02 : 01 – $DPB1$*01 : 01	8	0.931	0.931
**Average**			0.919			0.920	0.929
**p-value**			0.063			0.060	0.0612
**HLA-DQ molecules**							
$DQA1$*01 : 01 – $DQB1$*05 : 01	$DQA1$*01 : 02 – $DQB1$*06 : 02	0.055	0.708	$DQA1$*01 : 02 – $DQB1$*06 : 02	17	0.708	0.720

Continued on next page

Table S4 – continued from previous page

$DQA1{*}01:02-DQB1{*}06:02$	$DQA1{*}01:01-DQB1{*}05:01$	0.055	0.515	$DQA1{*}01:01-DQB1{*}05:01$	17	0.515	0.684
$DQA1{*}03:01-DQB1{*}03:02$	$DQA1{*}05:01-DQB1{*}02:01$	0.030	0.719	$DQA1{*}05:01-DQB1{*}03:01$	7	0.581	0.651
$DQA1{*}04:01-DQB1{*}04:02$	$DQA1{*}03:01-DQB1{*}03:02$	0.044	0.763	$DQA1{*}03:01-DQB1{*}03:02$	11	0.763	0.728
$DQA1{*}05:01-DQB1{*}02:01$	$DQA1{*}03:01-DQB1{*}03:02$	0.066	0.749	$DQA1{*}03:01-DQB1{*}03:02$	19	0.749	0.687
$DQA1{*}05:01-DQB1{*}03:01$	$DQA1{*}03:01-DQB1{*}03:02$	0.030	0.653	$DQA1{*}03:01-DQB1{*}03:02$	7	0.653	0.619
**Average**			0.685			0.662	0.682
**p-value**			0.0319			0.030	0.319

Table S4: The first column gives the name of the query allele. **NN** allele and **H** allele denote the nearest neighbour allele that is the most similar and has th shortest distance to the allele in query among all other alleles in the training set, to nearest neighbour and Hamming distance approaches, respectively corresponding to the query allele. Likewise **NN** distance and **H** distance give the distance measurement between the two alleles calculated as described in Section 3.3 in the main text. The columns **NN AUC**, **H AUC** and **LOO AUC** show the prediction performance in terms of Area Under Curve-(AUC) Fawcett (2006) for nearest neighbour, H distance and LOO approaches, respectively.

REFERENCES

Friedman J, Hastie T, Tibshirani R. Regularization Paths for Generalized Linear Models via Coordinate Descent. *Journal of Statistical Software* **33** (2010) 1–22.

Andreatta M, Karosiene E, Rasmussen M, Stryhn A, Buus S, Nielsen M. Accurate pan-specific prediction of peptide-MHC class II binding affinity with improved binding core identification. *Immunogenetics* **67** (2015) 641–650. doi:10.1007/s00251-015-0873-y.

Zhang L, Chen Y, Wong HS, Zhou S, Mamitsuka H, Zhu S. TEPITOPEpan: Extending TEPITOPE for Peptide Binding Prediction Covering over 700 HLA-DR Molecules. *PLoS One* **7** (2012). doi:10.1371/journal.pone.0030483.

Zhao Li YZGPJT, Guo F. A Novel Peptide Binding Prediction Approach for HLA-DR Molecule Based on Sequence and Structural Information. *BioMed Research International* **2016** (2016). doi:10.1155/2016/3832176.

Fawcett T. An introduction to ROC analysis. *Pattern Recognition Letters* (2006). doi:10.1016/j.patrec.2005.10.010.

Degoot AM, Chirove F, Ndifon W. A Biophysical Model for Predictions of Peptide: HLA-DR Interactions Based on Genomic and Structural Data (*under review*). *BMC Bioinformatics* (2017).

Hollander M, Wolfe DA. *Nonparametric Statistical Methods* (John Wiley & Sons) (1999), 68–75 .
